# Angiotensin (1-7) Attenuates Sepsis-Induced Acute Kidney Injury by Regulating the NF-κB Pathway

**DOI:** 10.3389/fphar.2021.601909

**Published:** 2021-03-04

**Authors:** Ying Zhu, Daliang Xu, Fang Deng, Yonglin Yan, Jian Li, Chenyu Zhang, Jing Chu

**Affiliations:** ^1^ Department of Nephrology, Anhui Provincial Children’s Hospital, Hefei, China; ^2^ Department of Clinical Laboratory, Anhui Provincial Children’s Hospital, Hefei, China; ^3^ Department of Pathology, Anhui Provincial Children’s Hospital, Hefei, China

**Keywords:** sepsis, acute kidney injury, renin-angiotensin system, nuclear factor-κB, angiotensin (1–7)

## Abstract

This study explores the protective mechanism of angiotensin (1-7) [Ang-(1-7)] on kidneys by examining its effects on renal histomorphology, inflammatory response, oxidative stress, and NF-κB signaling in mice suffering from sepsis-induced acute kidney injury. A sepsis-induced acute kidney injury mouse model was established by intracervically injecting lipopolysaccharides (LPS group), followed by the administration of Ang-(1-7) [LPS + Ang-(1-7) group]. The serum levels of urea nitrogen, creatinine and cystatin. c were measured with an automatic biochemical analyzer, and changes in proinflammatory cytokines and angiotensin II (Ang II) in the serum and kidneys were quantified by enzyme-linked immunosorbent assays. Changes in oxidative stress indices in the renal cortex were detected by colorimetry. The localization of Ang II in kidneys was examined by immunohistochemistry. Western blotting was used to examine phosphorylated NF-κB-p65 and IκBα levels in kidneys. Compared with the control group, the serum levels of urea nitrogen, creatinine and cystatin. c were increased, whereas the levels of Ang II, TNFα, IL-1β, IL-6, and malondialdehyde (mda) were increased significantly. The levels of Ang II and phosphorylated NF-κB-p65 were elevated in kidneys, whereas the levels of superoxide dismutase (sod), Total antioxidative capacity (TAOC), and inhibitor of NF-κB (IκBα) were reduced in the LPS group (*p* < 0.05). Pathological damage was also observed in kidneys of LPS-group mice. In Pearson correlation analysis, there was a positive correlation between Ang II and phosphorylated NF-κB-p65 levels, and a negative correlation between Ang II and IκBα levels (*p* < 0.05). After the application of Ang-(1-7), the levels of urea nitrogen, creatinine, cystatin. c, Ang II, TNFα, IL-1β, IL-6, and mda, as well as the expression of Ang II and phosphorylated NF-κB-p65 in kidneys of LPS + Ang-(1-7)-group mice, were lower than those in kidneys of LPS-group mice, but the levels of sod, TAOC, and IκBα were higher than those of LPS-group mice (*p* < 0.05). Pathological changes were less severe in mice of the LPS + Ang-(1-7) group. Overall, Ang-(1-7) can decrease the Ang II level, inhibit NF-κB signaling, reduce the inflammatory response, decrease oxidative stress, and mitigate sepsis-associated acute kidney injury.

## Introduction

Sepsis-associated acute kidney injury, a condition in which renal function declines rapidly in a short period of time, is characterized by pathophysiological changes that disrupt renal cortex and medullary blood flow and cause tubule necrosis (Alobaidi et al., 2015; Santos et al., 2018). Sepsis-associated acute kidney injury can also affect many other organs and systems. The incidence of sepsis-associated acute kidney injury increases each year, and the number of cases that progress to chronic renal failure and subsequently end-stage renal disease has increased the mortality rate (Bagshaw et al., 2007; Mehta et al., 2016; Fiorentino et al., 2018). Therefore, it is important to identify effective renal protective agents to eliminate or alleviate acute kidney injury and to reduce the mortality rate.

The mice model of sepsis induced by injection of lipopolysaccharides (LPS) is a common animal model for sepsis research, through which bacterial endotoxin induces animal to produce pathophysiological responses. This is similar to the development of sepsis in humans. Nuclear factor-B (NF-κB) is activated by the toll-like receptor 4 (TLR4) on the membrane surface of the target cells, which leads to excessive systemic inflammatory response and oxidative stress. In this way, the physiological action of LPS is achieved (Smith et al., 2015; Mir et al., 2018; Islam et al., 2019; Niu et al., 2019).

The activation of renin-angiotensin system and an elevated angiotensin II (Ang II) level are involved in various forms of acute kidney injury (Sato et al., 2012; Zhang et al., 2016; Chou et al., 2017; Chou et al., 2018; Safari et al., 2019; Sharma et al., 2019). In addition, Ang II acts as an effector molecule of renin-angiotensin system and also as an inflammatory factor that binds to G protein-coupled receptor (AT1R), leading to sustained contraction of renal vessels, activating TLR4 through Rho kinase pathway. Then it stimulates the activation of NF-κB in mesangial cellsand triggers inflammation and lipid peroxidation cascade, which are likely to cause severe renal injury (Wolf et al., 2002; Wolf et al., 2006; Kim et al., 2012; Kumar et al., 2015; Cao et al., 2016; Jin et al., 2019; Liu et al., 2019). Therefore, the treatment strategies for acute kidney injury involve using angiotensin converting enzyme inhibitor (ACEi) and angiotensin receptor blocker (ARB) (Chou et al., 2017; Chou et al., 2018; Safari et al., 2019). Nevertheless, ACEi and ARBs both cause vasodilation of the renal efferent arterioles and result in reduction of glomerular filtration pressure. During hypovolemia, the reduced efferent vascular tone lowers glomerular filtration rate and ultimately promoting AKI. The good news is that ACE2-Ang-(1-7)-Mas axis, as a newly discovered branch of renin-angiotensin system, has been gradually understood (Santos et al., 2018; Safari et al., 2019).

In kidney, angiotensin 1-7 [Ang-(1-7)] is predominantly formed in the renal cortex (Joyner et al., 2007). The concentrations of Ang-(1-7) and AngII are comparable in kidney, and Ang-(1-7) is detectable in human urine (Grobe et al., 2012). Ang-(1-7) has multiple biological effects when acting on Mas receptors, such as vasodilation, lowering blood pressure, as well as antithrombotic, anti-fibrotic, anti-inflammatory, and antioxidant effects, which are contrary to the ACE-Ang II-AT1 axis (Santos et al., 2018; Zhang et al., 2018). Animal experiments have shown that the ACE2-Ang-(1-7)-Mas axis plays a vital role in preventing LPS-induced acute lung injury by inhibiting the NF-kB pathway (Li et al., 2015; Li et al., 2016; Chen et al., 2018). Considerable evidence has revealed that ACE2-Ang-(1-7)-Mas axis plays an important role in improving renal function, reducing proteinuria, increasing creatinine clearance, and improving renal structure (Zhang et al., 2010; Giani et al., 2011). Moreover, Ang-(1-7) and its novel analogue have shown their promising therapeutic effects against acute kidney injury (Zhang et al., 2019), while their *in vivo* function in kidney injury caused by sepsis remains unclear. Therefore, in this study, sepsis-associated acute kidney injury mice were induced by LPS as an experimental model. Through exogenously providing Ang-(1-7), we aim to observe if Ang-(1-7) can mitigate the renal tissue injury, inflammatory responses, and oxidative stress in sepsis-associated acute kidney injury. We also designated to preliminarily explore the underlying potential signaling pathways, so as to provide a new theoretical and experimental basis for the prevention and treatment of sepsis-associated acute kidney injury.

## Methods

### Materials

LPS was purchased from Sigma-Aldrich (St. Louis, MO, United Ststes). Ang-(1-7) was purchased from Gill Biochemistry (Shanghai, China). Ang II enzyme-linked immunosorbent assay (ELISA) kit, Ang II polyclonal antibody, TNFα, IL-1β, and IL-6 were purchased from Ray Biotech (Guangzhou, China). rabbit anti-Phospho-NF-κB-p65 (Ser536) and rabbit anti-IκBα were purchased from Cell Signaling Technology (Boston, United Ststes). Glyceraldehyde-3-phosphate dehydrogenase (GAPDH) and bovine serum albumin (BSA) were obtained from Jiangsu Kaiji Biotechnology (Nanjing, China). Malondialdehyde (mda), superoxide dismutase (sod), and total antioxidant capacity (TAOC) detection kits were provided by Nanjing Institute of Bioengineering (Nanjing, China).

### Animals and Establishment of the Model

Eighty C57BL/6 male mice, aged 6–8 weeks and weighing 25–32 g (provided by Anhui Medical University, Anhui, China), were used. After 2 weeks of adaptive feeding, mice were randomly divided into four groups (*n* = 20 per group). Each mouse was anesthetized and a micro-osmotic pump was implanted scapula-subcutaneously. For the control group, saline was administered for 15 days. For the Ang-(1-7) group, Ang-(1-7) was pumped into the body at 144 μg/kg per day (Yamada et al., 1998; Giani et al., 2012; Zheng et al., 2015). For the sepsis group (LPS group), saline was administered for 15 days, followed by a single jugular vein injection of LPS at 10 mg/kg on day 14 (Ruetten et al., 1996; Gupta et al., 2007; Chen et al., 2019). For the sepsis and Ang-(1-7) [LPS + Ang-(1-7) group] group, Ang-(1-7) was administered for 15 days, followed by a single intracervical injection of LPS at 10 mg/kg on day 14.

All mice were housed under conditions of 12-h light: 12-h dark, a room temperature of 25 ± 1 °C, and a relative humidity of 50–70%. Food and water were available *ad libitum*. Mice were sacrificed, and tissues were harvested.

### Animal Handling and Sampling

The survival rate of mice in LPS and LPS + Ang-(1-7) groups was determined, and sampling was carried out as indicated. On day 15, mice in control and Ang-(1-7) groups were anesthetized with choral hydrate at 0.35 ml/100 g. Approximately 4 ml of blood was collected from the sinus vein and serum was prepared. Serum samples were divided and stored at −80 °C, and the levels of urea nitrogen, creatinine, cystatin.c, TNFα, IL-1, IL-6, and Ang II were measured. Kidneys were rinsed in prechilled saline, followed by the removal of capsules, connective tissues, and blood vessels. The renal cortex was removed, and the upper two-thirds of right kidneys were placed in liquid nitrogen overnight. Specimens were stored at −80 °C, and the levels of phosphorylated NF-κB-p65 and IκBα were measured. To prepare 10% homogenates, approximately 1 g of kidney (lower one-thirds of right kidneys, middle one-thirds of left kidneys) was removed, combined with 9 ml of saline, and homogenized using a glass homogenizer. Homogenates were centrifuged at 4 °C for 10 min at 2,500 rpm, and supernatants were stored at 4 °C. Samples that were not immediately assayed were stored at −80 °C for no more than 10 days. The levels of TNFα, IL-1β, IL-6, mda, sod, and TAOC were measured in these samples. The upper one-thirds of left kidneys were fixed in 10% formaldehyde and processed for paraffin embedding. Sections were stained with hematoxylin and eosin, and Ang II was localized by immunohistochemistry. The lower one-thirds of left kidneys were fixed in 2.5% glutaraldehyde for electron microscopy.

### Determination of Renal Function in Mice

Approximately 0.2 ml of serum was collected and sent to the laboratory of the Anhui Provincial Children’s Hospital for measurement of urea nitrogen (by urease—glutamate dehydrogenase method), creatinine (sarcosine oxidase method) and cystatin.c (Immunoturbidimetry) levels with an automatic biochemical analyzer.

### Pathological Observation of Kidneys

The kidney tissues from mice were embedded into paraffin, followed by staining with hematoxylin and eosin (HE), Periodic Acid-Schiff stain (PAS). Pathological changes in the glomerulus, renal tubules, renal interstitium, and renal vasculature were examined using a LEICA (DM2000, Germany) optical microscope.

### Preparation and Observation of Kidney Specimens by Electron Microscopy

Semi-thin sections were prepared with an ultramicrotome (LKB-m, Swedish) and stained with uranyl acetate and lead citrate. Photomicrographs were obtained using a JEM-1400 Flash microscope (JEOL Co., Ltd., Japan).

### Measurement of the Ang II Level in Serum and Kidneys

The Ang II level was measured by a double antibody sandwich enzyme-linked immunosorbent assay (ELISA). Using the SABC method, 1 µm-thick kidney paraffin sections were dewaxed and rehydrated using a graded alcohol series. Endogenous peroxidase activity was blocked with 3% hydrogen peroxide, followed by antigen retrieval. Calf serum was used as the blocking agent. After incubating with the primary antibody (Ang II, 1:100), a secondary antibody was used, followed by streptavidin application (SABC solution), color development with DAB, and staining with hematoxylin. PBS served as the negative control. Image analysis was used to determine the staining intensity of Ang II. Two sections were randomly selected from each animal, and five fields of view were evaluated to calculate the average optical density, which was the relative level of Ang II expression.

### Detection of Inflammatory Index

The levels of TNFα, IL-1β, and IL-6 in serum and kidneys of mice were measured by ELISA. The optical density was detected at 450 nm and the protein concentration was obtained from the absorbance curves generated following standards for recombinant proteins. The assays were performed following the manufacturer’s protocols.

### Detection of Oxidative Stress Indices

Homogenate of kidney tissue was prepared as described previously, and the levels of oxidative stress indices were evaluated using the indicated commercial kits. The mda level in homogenates was measured by the thiobarbituric acid method using the mda determination kit. The sod level was measured by the hydroxylamine method using the sod determination kit. The TAOC level was measured by the colorimetric method using the TAOC determination kit. Renal cortex protein concentrations were estimated by the Coomassie blue method as a correction. All procedures were performed as described in the assay kit.

### Western Blotting to Examine the Levels of Phosphorylated NF-κB-p65 and IκBα in Kidneys

Equal amounts of total protein were separated by sodium dodecyl sulfate polyacrylamide gel electrophoresis. Proteins were transferred onto polyvinylidene difluoride membranes, followed by blocking with 5% bovine serum albumin for 2 h. Membranes were incubated with the indicated primary antibodies. On the following day, membranes were washed with Tris-buffered saline containing 0.1% Tween-20 and incubated with a secondary antibody. Protein bands were developed by enhanced chemiluminescence and imaged with a gel imager. Protein analysis was performed with Gel-Pro32 software.

### Statistical Methods

SPSS 21.0 software (IBM, Armonk, NY, United States) was used to analyze the data. Measurement data were presented as means ± standard deviation. One-way analysis of variance (ANOVA) was used to compare data between groups. The homogeneity test of variance was also performed. For equal variance, the least significant difference (LSD) was used to analyze differences between groups. For unequal variance, Dunnett’s T3 was used. Numerical data were examined by the Chi Square test (*χ* ^2^). Pearson correlation analysis was used for variables with correlation. *p*-values less than 0.05 were statistically significant.

## Results

### Ang-(1-7) Affects the Survival Rate of Mice

**FIGURE 1:**
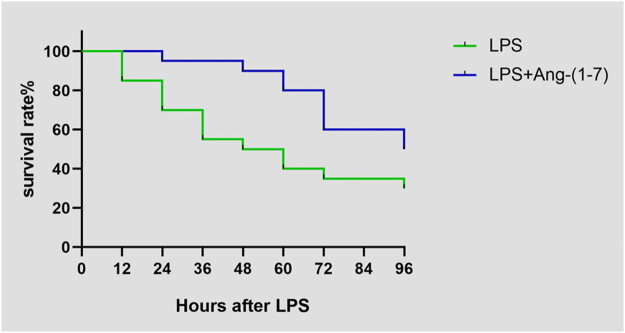
Effect of Ang-(1–7) on animal survival after LPS treatment. Animal survival was recorded at indicated time points.

The survival rate of mice injected with LPS on day 4 was 30% (6/20), whereas that of mice injected with both LPS and Ang-(1-7) was 50% (10/20). There was a significant difference between the two groups (*χ* ^2^ = 5.590, *p* = 0.0181) (Figure 1fig1).

### Changes in Renal Function in Sepsis-Associated Acute Kidney Injury

No differences were observed in the levels of urea nitrogen, creatinine and cystatin.c between control and Ang-(1-7) groups (*p* > 0.05). However, their levels of urea nitrogen, creatinine and cystatin.c were significantly increased in LPS and LPS + Ang-(1-7) groups (*p* < 0.05). Overall, the levels of both indices from the highest to the lowest were: LPS group > LPS + Ang-(1-7) group > control group > Ang-(1-7) group (Table 1).

**TABLE 1:** Changes in renal function and the Ang II level in mice.

Group	Urea nitrogen (mmol/L)	Creatinine (μmol/L)	Cystatin.c (mg/L)	Serum ang II (ng/ml)	Positive ang II expression in kidneys
LPS + Ang-(1-7)	22.79 ± 2.98*#	75.29 ± 7.68*#	1.96 ± 0.28*#	2.35 ± 0.18*#	1.01 ± 0.05*#
LPS	32.70 ± 4.80*	112.51 ± 9.12*	2.51 ± 0.79*	2.82 ± 0.26*	1.25 ± 0.05*
Ang-(1-7)	4.93 ± 1.05	26.50 ± 3.41	0.90 ± 0.22	1.74 ± 0.10	0.49 ± 0.03
Control	4.95 ± 0.98	26.69 ± 3.53	0.91 ± 0.24	1.76 ± 0.11	0.50 ± 0.03
*F/P* value	446.90/<0.01	837.57/<0.01	63.08/<0.01	176.65/<0.01	1,631.54/<0.01

*, compared with the control group, *p* < 0.01; #, compared with the LPS group, *p* < 0.05.

### Pathological Changes in Kidneys in Sepsis-Associated Acute Kidney Injury

**FIGURE 2:**
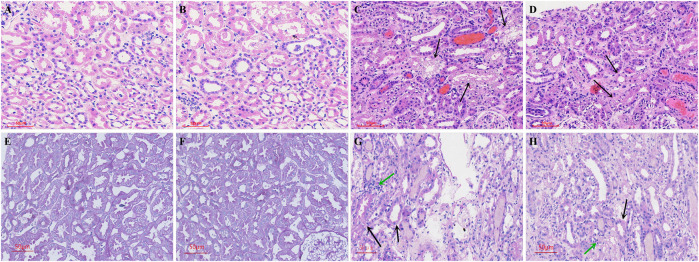
Pathological changes in kidneys from the four groups of mice. Cross-sections were stained with HE and PAS stain. HE stain: A–D. **(A)** Control group. **(B)** Ang-(1–7) group. **(C)** LPS group. The arrows indicate swelling of epithelial cells of renal tubules, vacuolar degeneration, and necrosis in the renal cortex and interstitium. **(D)** LPS + Ang-(1–7) group. Pathological damage was less severe in the LPS + Ang-(1–7) group than that in the LPS group. PAS stain: E–H. **(E)** Control group. **(F)** Ang-(1–7) group. **(G)** LPS group. The black arrows indicate the insoluble purple-red complex in the renal tubule cytoplasm, the green arrows indicate inflammatory cell infiltration. **(H)** LPS + Ang-(1–7) group. The black arrows represent the decrease of insoluble purple-red complex in the renal tubule cytoplasm, the green arrow represents reduced inflammation infiltration in the LPS + Ang-(1–7) group. Bars in A–H = 50 µm.

**FIGURE 3:**
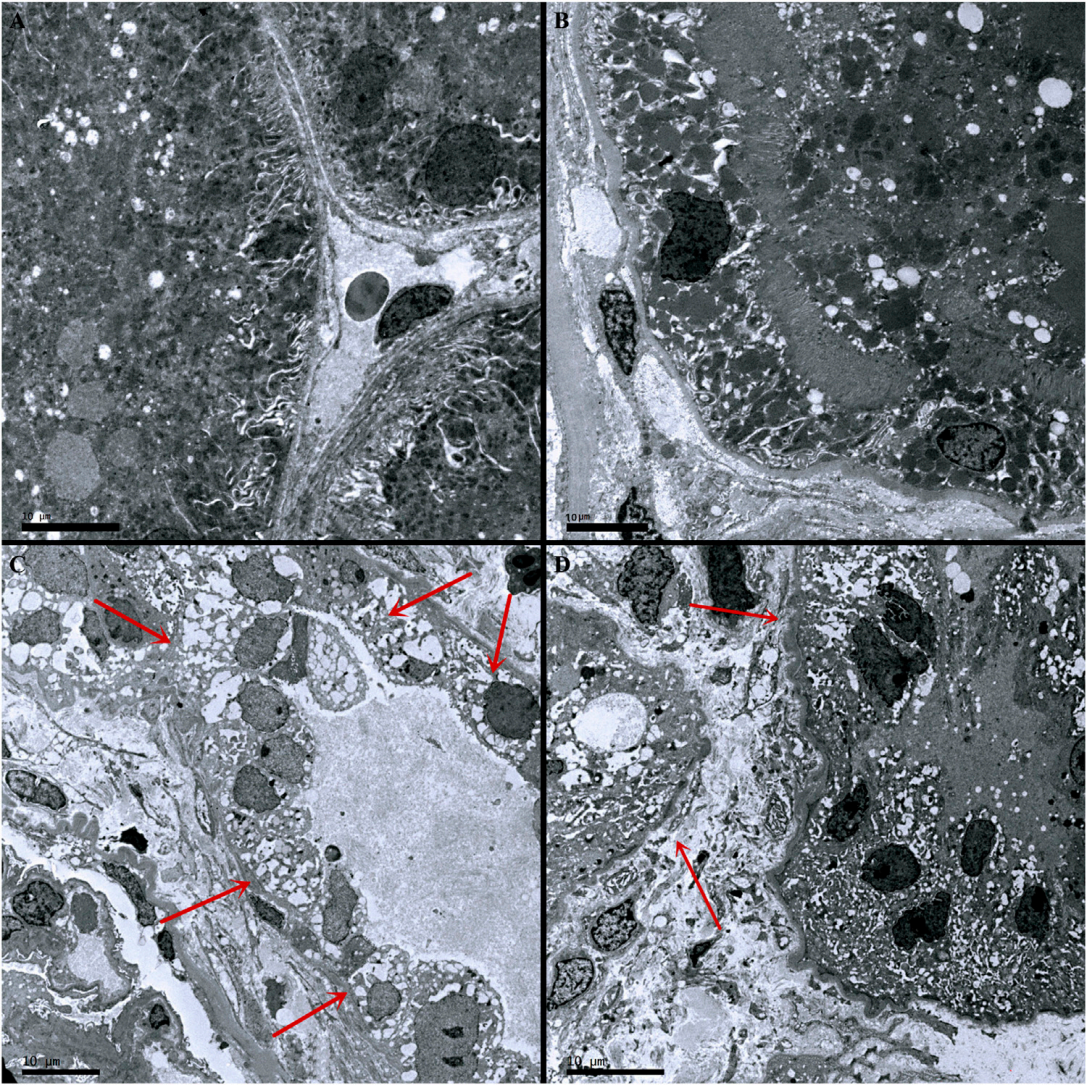
Pathological changes in kidneys from the four groups of mice. **(A)** Control group. **(B)** Ang-(1–7) group. **(C)** LPS group. The microvilli of epithelial cells in renal tubules disappeared and part of the epithelium disintegrated. **(D)** LPS + Ang-(1–7) group. Red arrows indicate the pathological changes of renal tubular epithelial cells. Some mitochondria in epithelial cells of renal tubules swelled, and microvilli shedding and loss was noted. Pathological damage was less severe in the LPS + Ang-(1–7) group than that in the LPS group. Bars in A–D = 10 µm.

Compared with the control group, there was no inflammatory cell infiltration into the renal cortex and interstitium, and no pathological changes in epithelial cells of renal tubules in the Ang-(1-7) group. However, there was inflammatory cell infiltration into the renal cortex and interstitium in LPS and LPS + Ang-(1-7) groups, and most epithelial cells of renal tubules showed swelling, vacuolar degeneration, and necrosis. By electron microscopy, we observed the disappearance of microvilli and the disintegration of the epithelium. Pathological damage in the LPS + Ang-(1-7) group was less severe than that in the LPS group. No pathological changes were observed in the glomerulus of all groups (Figures 2, 3).

### Changes in the Ang II Level in Serum and Kidneys in Sepsis-Associated Acute Kidney Injury

No significant difference (*p* > 0.05) was noted in the serum Ang II level between control and Ang-(1-7) groups. However, the level was higher (*p* < 0.01) in the LPS + Ang-(1-7) group than that in the control group, whereas it was lower in the LPS + Ang-(1-7) group than that in the LPS group (*p* < 0.01) (Table 1).

**FIGURE 4:**
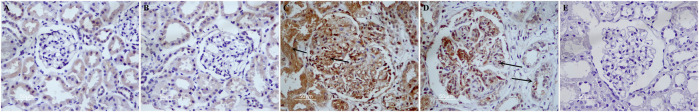
Localization of Ang II in kidneys from the four groups of mice. Immunohistochemistry was performed as described in the Materials and Methods. **(A)** Control group. In the control group, Ang II is scattered in the cytoplasm of renal tubular epithelial cells, and the expression of glomerulus is very little. **(B)** Ang-(1–7) group. **(C)** LPS group. Ang II positive results are brown particles. The black arrows indicate positive expression. **(D)** LPS + Ang-(1–7) group. The black arrows indicate positive expression. The expression intensity and range of Ang II were significantly reduced compared with the LPS group. **(E)** Negative control group. Bars in A–E = 50 µm.

Ang II expression in kidneys from all groups of mice was examined. Compared with the control group, there was no difference in Ang II expression in the Ang-(1-7) group (*p* > 0.05). However, Ang II expression was increased in LPS and LPS + Ang-(1-7) groups (*p* < 0.01). Compared with the LPS group, the intensity and area of the Ang II immunohistochemical signal was greatly reduced in the LPS + Ang-(1-7) group (*p* < 0.01) (Table 1; Figure 4).

### Changes in Inflammatory Cytokines in Sepsis-Associated Acute Kidney Injury

No differences in the levels of TNFα, IL-1β, and IL-6 in serum and kidneys were detected in control and Ang-(1-7) groups (*p* > 0.05). However, the levels of these proinflammatory cytokines were significantly increased in LPS and LPS + Ang-(1-7) groups (*p* < 0.01) compared with the control group, with levels in the LPS + Ang-(1-7) group lower than those in the LPS group (*p* < 0.01) (Table 2).

**TABLE 2:** Changes in inflammatory marker levels in mice.

**Group**	**Serum**	**Renal**	**Serum**	**Renal**	**Serum**	**Renal**
	**TNFα**	**TNFα**	**IL-1β**	**IL-1β**	**IL-6**	**IL-6**
	**(pg/ml)**	**(pg/ml)**	**(pg/ml)**	**(pg/ml)**	**(pg/ml)**	**(pg/ml)**
LPS + Ang-(1-7)	153.31 ± 9.51*^#^	691.95 ± 44.38*^#^	76.20 ± 7.12*^#^	133.35 ± 15.70*^#^	493.01 ± 66.20*^#^	819.61 ± 83.43*^#^
LPS	216.94 ± 24.98*	951.08 ± 94.79*	105.95 ± 11.00*	198.01 ± 21.12*	834.80 ± 93.62*	1,530.32 ± 113.32*
Ang-(1-7)	1.11 ± 1.86	1.73 ± 2.46	2.31 ± 3.26	4 0.64 ± 6.56	0.89 ± 1.40	4.77 ± 6.76
Control	1.30 ± 1.96	2.14 ± 2.71	2.41 ± 3.30	4 0.86 ± 6.68	1 0.01 ± 1.34	5 0.49 ± 7.19
*F/P* value	1,325.07/<0.01	1714.78/<0.01	1,146.22/<0.01	956.51/<0.01	1,009.62/<0.01	2,172.32/<0.01

*, compared with the control group, *p* < 0.01; #, compared with the LPS group, *p* < 0.01

### Changes of Kidney Weight and Oxidative Stress Indices

No significant difference in kidney weight was observed between Ang-(1-7) and control groups, as well as no differences in the levels of mda, sod, and TAOC between these groups (*p* > 0.05). However, significantly increased kidney weight, upregulated mda level, and downregulated sod and TAOC levels were observed in both the LPS and LPS + Ang-(1-7) groups (*p* < 0.01). Compared with the LPS group, the kidney weight and the mda level in the LPS + Ang-(1-7) group were lower, whereas the levels of sod and TAOC were significantly higher (*p* < 0.01) (Table 3).

**TABLE 3:** Changes in kidney weight and oxidative stress indices in mice.

Group	Right kidney weight/Body weight (×10^–3^)	mda (nmol/mg pr)	TAOC (U/mg pr)	Sod (U/mg pr)
LPS + Ang-(1-7)	5.32 ± 0.20*^#^	1.80 ± 0.12*^#^	1.62 ± 0.08*^#^	419.31 ± 19.80*^#^
LPS	6.26 ± 0.27*	3.67 ± 0.27*	0.91 ± 0.08*	313.01 ± 11.10*
Ang-(1-7)	4.45 ± 0.19	0.98 ± 0.15	2.37 ± 0.14	500.79 ± 22.65
Control	4.43 ± 0.15	1.02 ± 0.16	2.37 ± 0.16	506.73 ± 19.97
*F/P* value	353.44/<0.01	922.84/<0.01	681.07/<0.01	459.82/<0.01

*, compared with the control group, *p* < 0.01; #, compared with the LPS group, *p* < 0.01

### Changes in Phosphorylated NF-κB-p65 and IκBα in Sepsis-Associated Acute Kidney Injury

**FIGURE 5:**
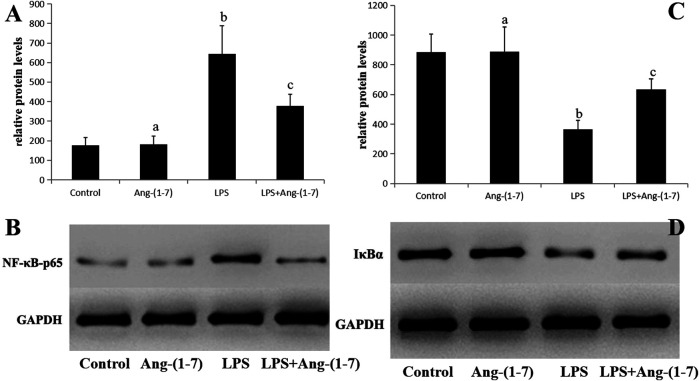
Changes in phosphorylated NF-κB-p65 and IκBα in sepsis-associated acute kidney injury. **(A)** Phosphorylated NF-κB-p65 protein level in kidneys from the four groups of mice. The letters a, b, and c represent statistical difference among groups at *p* < 0.05. **(B)** Phosphorylated NF-κB-p65 protein level in kidneys from the four groups of mice. **(C)** IκBα protein level in kidneys from the four groups of mice. The letters a, b, and c represent statistical difference among groups at *p* < 0.05. **(D)** IκBα protein level in kidneys from the four groups of mice.

No significant differences in the levels of phosphorylated NF-κB-p65 and IκBα in the Ang-(1-7) group (*p* > 0.05). However, the phosphorylated NF-κB-p65 level increased and that of IκBα decreased in LPS and LPS + Ang-(1-7) groups, with the phosphorylated NF-κB-p65 level in the LPS + Ang-(1-7) group lower than that in the LPS group and the IκBα level in the LPS + Ang-(1–7) group higher than that in the LPS group (*p* < 0.05) (Figure 5).

### Correlations Between the Levels of Phosphorylated NF-κB-p65 and IκBα and the Level of Ang II in the Serum and Kidneys in Sepsis-Associated Acute Kidney Injury

**FIGURE 6:**
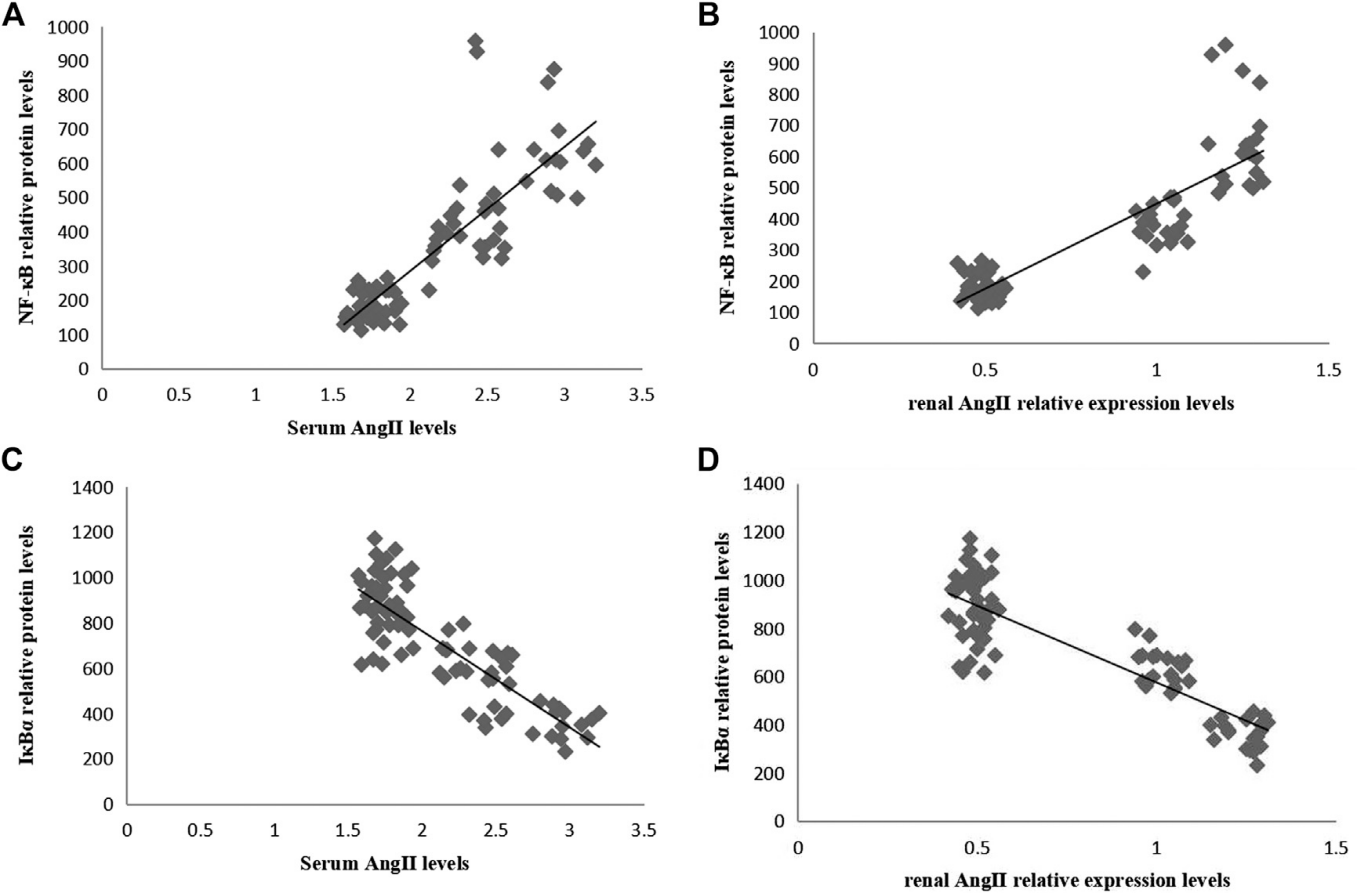
Correlation analyses between the relative protein levels of NF-κB-p65 and IκBα and the level of Ang II in serum and kidneys. **(A)** Correlation between the serum Ang II level and the relative protein level of renal NF-κB-p65 (*r* = 0.844, *p* = 0.000). **(B)** Correlation between renal Ang II level and relative protein level of renal NF-κB-p65 (*r* = 0.877, *p* = 0.000). **(C)** Correlation between serum Ang II level and relative protein level of renal IκBα (*r* = −0.847, *p* = 0.000). **(D)** Correlation between renal Ang II level and relative protein level of renal IκBα (*r* = −0.873, *p* = 0.000).

In Pearson correlation analysis, there was a positive correlation between Ang II and phosphorylated NF-κB-p65 levels (Figures 6A,B), and a negative correlation between Ang II and IκBα levels (Figures 6C,D).

## Discussion

In sepsis, the expression of vasoactive hormone receptors is reduced, which further weakens the vascular bed, induces peripheral vasodilation, excites the sympathetic nervous system, and activates renin-angiotensin system, thereby causing blood vessels to constrict continuously, spasm to glomerular afferent arteries, decrease kidney blood flow, and induce hypoxic-ischemic conditions, finally leading to acute kidney injury (Sharma et al., 2019). A close relationship between sepsis-associated acute kidney injury and renin-angiotensin system was noticed. The mice were initially in good health condition. After the injection of LPS, the mice experienced less food and water consumption, reduced physical activities, and exhibited fluffy hair. Several of the mice died from 12 h to 24 h. We observed significantly increased serum creatinine, urea nitrogen, cystatin.c and mortality rate in the LPS-group mice. On the contrary, treatment with Ang-(1-7) resulted in a significant decrease of these parameters. Through histopathological examinations, we observed that the mice in the control group had normal kidney tissues, while renal inflammation and tubular damage were found in the mice of the LPS-group mice had. However, such a damage was mitigated in the mice of the Ang-(1-7) intervention group, which indicates that Ang-(1-7) possesses nephron-protective effect against LPS toxicity. Furthermore, immunohistochemical examinations detected that LPS induced a significantly upregulation of Ang II in the kidney tissue, and Ang-(1-7) could counteract the aberrant Ang II expression in kidney. Based on *in vivo* studies, we found that Ang-(1-7) can reduce the enlarged mesangial region, alleviate inflammation and fibrosis, and reduce oxidative stress in mice with diabetic nephropathy; the benefits of Ang-(1-7) were better than those of the ACEi (Mori et al., 2014; Zhang et al., 2015). Recent research found that Ang-(1-7) alone or in combination with losartan could protect the kidney against Ischemia/reperfusion (I/R) damage (Safari et al., 2019). Our previous studies have found that Ang II could promote human glomerular endothelial cell apoptosis, facilitate the production of reactive oxygen species, and increase the expression of inflammatory factors, while they were all inhibited by Ang-(1-7). The addition of Ang-(1-7) inhibited the damage of human glomerular endothelial cells induced by Ang II in a dose-dependent manner, while the protective effect of Ang-(1-7) was nearly completely counteracted by antagonist A779 (Zhang et al., 2018). These findings demonstrate that Ang-(1-7) can be prospectively applied for treating sepsis-associated acute kidney injury.

LPS was previously reported to induce the production of TNFα and IL-6 in kidneys of normal rats, and this effect was likely to be aggravated after renal intravenous administration of Ang II (Niimi et al., 2002). LPS stimulates NF-κB, mitogen-activated protein kinase (MAPK) family, and other signals to transmit into cells through binding with CD14 and TLR4, inducing the release of a large number of inflammatory factors, such as TNF-α, IFN-γ, IL-1, IL-6, and IL-12 (Smith et al., 2015; Patel et al., 2019; Zhou et al., 2020). These inflammatory factors are generally signal molecules and effectors that directly result in “inflammatory storm” through the NF-κB pathway, leading to cell damage. In this study, LPS significantly induced the secretion of pro-inflammatory cytokines TNF-α, IL-6, and IL-1β in kidney tissues. However, Ang-(1-7) significantly reduced their production in acute kidney injury induced by LPS, which suggests that Ang-(1-7) can mitigate kidney damage through its anti-inflammatory activity.

Oxidative stress is also involved in the pathogenesis of LPS-induced nephrotoxicity (Chen et al., 2019). In this study, the mda level was increased in the LPS group compared with that in the control group. Mda is a product from lipid peroxidation generated by oxygen free radicals, which attacks cell membranes. It can indirectly reflect the degree of endothelial cell damage, and is widely regarded as an indicator for monitoring the level of oxidative stress (Chen et al., 2019). We observed that LPS not only accelerated the formation of cytotoxic mda mediated by lipid peroxidation, but also inhibited renal antioxidant capacity. During oxidative stress, the body scavenges free radicals through producing antioxidant enzymes such as sod and glutathione peroxidase (Poljsak et al., 2013), while TAOC reflects the total antioxidant capacity of tissues. Ang-(1-7) significantly reduced mda level, restored sod and TAOC levels in renal tissues, and alleviated oxidative renal injury. As a matter of fact, during oxidative stress, the reactive oxygen species activate TLR2 and TLR4 and mediate the NF-κB pathway, regulating pro-inflammatory cytokine secretion, inducing inducible nitric oxide synthase and cyclooxygenase two expression (Niu et al., 2019), which leads to decreased renal blood flow, cell membrane damage, inhibition of mitochondrial function, and thus initiating apoptotic processes.

Thus, NF-κB activation is believed to be a key factor in LPS-induced inflammation and oxidative stress in kidneys. NF-κB functions in nearly all cells and tissues. It is involved in various pathophysiological reactions in the body, such as immunity and inflammation, which serves as a key factor for initiating inflammatory responses and regulating gene transcription (Mulero et al., 2013). Therefore, we examined the expression levels of phosphorylated NF-κB-p65 and IκB proteins in the kidney and found that LPS can promote the degradation of IκB and the activity NF-κB, while Ang-(1-7) treatment can inhibit such an process and restore physiological balance. A previous study reported that perfusion of Ang II can activate NF-κB signaling in kidneys, accelerate inflammatory cell infiltration, and trigger oxidative stress, eventually resulting in the swelling of glomerular endothelial cells and necrosis of epithelial cells in renal tubules, which damages renal function (Ruiz-Ortega et al., 2001). We further assessed the correlation between renin-angiotensin system and NF-κB activity, and identified a positive correlation between the levels of Ang II and phosphorylated NF-κB-p65, and a negative correlation between the levels of Ang II and IκBα. Ang-(1-7) could inhibit LPS-induced NF-κB activation by down-regulating p65 levels. Therefore, we argue that Ang-(1-7) could alleviate sepsis-associated acute kidney injury by inhibiting the activation of Ang II, thereby blocking IκBα/NF-κB signaling and reducing inflammation and oxidative stress.

## Conclusion

In summary, our results demonstrated that Ang-(1-7) had a potential renal protective effect on the LPS mouse model. The nephron-protective effect of Ang-(1-7) may be achieved by inhibiting the production of pro-inflammatory cytokines, maintaining the redox balance, and suppressing the activation of NF-κB signal that is mediated by inflammation and oxidative stress. However, its specific molecular mechanism and interaction with Ang II needs to be further clarified. Besides, its functions need to be further investigated based on other acute kidney injury models.

## Data Availability

The original contributions presented in the study are included in the article/Supplementary Material, further inquiries can be directed to the corresponding authors.
